# Predictors for Estimating Scars’ Internalization in Victims with Post-Traumatic Scars versus Patients with Postsurgical Scars

**DOI:** 10.3390/healthcare10030550

**Published:** 2022-03-16

**Authors:** Gabriel Mihai Mekeres, Florica Voiţă-Mekereş, Cristina Tudoran, Camelia Liana Buhaş, Mariana Tudoran, Mariana Racoviţă, Nuţu Cristian Voiţă, Nicolae Ovidiu Pop, Mihai Marian

**Affiliations:** 1 Faculty of Medicine and Pharmacy, University of Oradea, Universitatii Street Nr. 1, 410087 Oradea, Romania; mekeres.gabriel.mihai@student.uoradea.ro (G.M.M.); racovita.mariana@student.uoradea.ro (M.R.); voita.nutucristian@student.uoradea.ro (N.C.V.); 2 County Clinical Emergency Hospital of Oradea, 410087 Oradea, Romania; cameliabuhas@uoradea.ro; 3 Department of Morphological Disciplines, Faculty of Medicine and Pharmacy, University of Oradea, 1 Universitatii Street, 410087 Oradea, Romania; 4 Department VII, Internal Medicine II, University of Medicine and Pharmacy “Victor Babes” Timisoara, E. Murgu Square, Nr. 2, 300041 Timisoara, Romania; tudoran.mariana@umft.ro; 5 Center of Molecular Research in Nephrology and Vascular Disease, Faculty of Medicine, University of Medicine and Pharmacy “Victor Babes” Timisoara, E. Murgu Square, Nr. 2, 300041 Timisoara, Romania; 6 County Emergency Hospital, L. Rebreanu Street, Nr. 156, 300723 Timisoara, Romania; 7 Department of Surgical, Faculty of Medicine and Pharmacy, University of Oradea, 1 Universitatii Street, 410087 Oradea, Romania; pop.nicolaeovidiu@didactic.uoradea.ro; 8 Psychology Department, Faculty of Socio-Humanistic Sciences, University of Oradea, 1 Universitatii Street, No. 3, 410087 Oradea, Romania; mmarian@uoradea.ro

**Keywords:** scars, mental suffering, internalization, psychometric methods

## Abstract

(1) Background: Scars are the consequence of physiological inherent healing processes of post-traumatic and surgical lesions with a psychological impact. Post-traumatic scarring may induce emotional and behavioral changes through social stigma. In this study we analyze the internalization of scars and their impact on hopelessness, depression, or the perception of social support in subjects with post-traumatic scars compared to people with surgical scars. (2) Methods: to research this suggested model, we analyzed data collected from 110 participants 61 women and 49 men, aged between 18 and 64 years; 55 participants had post-traumatically and 55 surgically acquired scars. They all were examined to assess the characteristics of scars, were asked to complete four psycho-social scales, and the results were compared. (3) Results: our results indicate that people with post-traumatic scars are oriented toward the internalization of scars, depending on their shape and size. We argue that hopelessness, appreciation of scars, age, and how scars are produced are important predictors of internalization. (4) Conclusions: the patient’s attitude toward the appearance of a scar is an indicator of how he/she will react in the future and it could predict the vulnerability to hopelessness. Finally, we nuance the impact of objective bodily harm on the psychological and moral suffering.

## 1. Introduction

The scars at the level of the skin represent a normal and inevitable process of healing of traumatic or surgical lesions, but except for the appearance of the skin, they also have a profound psychological impact [1,2]. Thus, people with scars try to integrate them into their own sense of self to gain psychological acceptance [3]. The visibility of scars acts as a mediator of psychological suffering, thus, hiding them improves maladaptive behavior and leads often to a return to normal functioning [4,5].

Unsightly morphological changes may occur in various circumstances being either post-traumatic (consequences of hitting, bodily injury, acts committed intentionally or through fault) or they can be acquired post-surgically [6,7,8]. Visible scars, especially those located on the face, have aesthetic and psychological consequences, such as increased anxiety and low self-esteem. The stigma due to the presence of scars is perceived depending on their appearance and location, so the affected people adapt by hiding them, they become less sociable, lower self-confidence, negatively affecting their personal and professional relationships as well as their leisure activities [9].

The traumatized person can be addressed to the forensic services for an initial evaluation of the injury, and in case of aesthetic consequences, for re-assessments after a longer period of time, which is necessary to finalize the appearance of the scar, the minimum recommended duration being six months in the case of a medium scar [10,11]. 

The increasing frequency of traumatic incidents leading to aesthetic damage requires new studies to develop more accurate methods for quantifying both, the morphology of scars and their psychosocial impact as well, potentially leading to a post-traumatic stress disorder. Post-traumatic scars may determine emotional and behavioral changes through social stigma and, in some cases, may have a triggering effect for re-experiencing the causative traumatic incident [12,13].

Progresses in the plastic and reconstructive surgery resulting in new techniques aim to remedy scarring secondary to traumas and surgery as well [14,15], especially of those located on the face, conduced to aesthetic improvements of scars appearance, but it is still necessary to analyze their psychosocial effects by using psychometric scales whose results are measured and reported according to the patient’s perspective [16]. 

Bodily harm can be evaluated, but for the elements of moral suffering there are no objective criteria. Evaluation scales have difficulty in assessing the role of mental suffering, given their process of development, modifying sometimes, the physiognomy or even the victim’s aesthetic perception of its own body [17]. 

The objective of this study is the analysis of the internalization of scars and their impact on the disposition or perception of social support in the case of people with surgical scars versus people with post-traumatic scars.

## 2. Materials and Methods

### 2.1. Study Population

In this study were included a total of 110 Participants, aged between 18 and 64 years, mean age 40.33 ± 13.53 years of which 61 were women and 49 men. The participants were distributed in two relatively homogeneous groups: the experimental group (*n* = 55) and c:ntrols (*n* = 55), as presented below.

- The experimental group included a number of 55 participants, 30 women and 25 men, aged between 18 and 64 years (mean age 39.98 years), selected during the first of September and first of November 2021, of the individuals who attended the Forensic Medicine Service of County Bihor, for forensic expertise to obtain a forensic certificate; other study participants were recruited from Emergency Unit of County Emergency Clinical Hospital, Oradea, while attending this service for other pathologies. Following the clinical exam and anamnesis, significant scars were observed, qualifying them for our study. Some other patients were addressed from the Plastic and Reconstructive Surgery Department of the County Emergency Clinical Hospital, Oradea. All these participants had post-traumatic scars: either after a road accident, work or household accident, or as a result of aggression.
- The control group included 55 participants, 31 women and 24 men, aged between 19 and 64 years (mean age 40.69 years) with scars acquired after surgery.

All participants, from the experimental and control group as well, were evaluated for eligibility and were asked to sign an inform consent, being afterwards included in the study. 

They were clinically examined to confirm the existence and characteristics of scars. 

### 2.2. Instruments

- Mekeres’ Psychosocial Internalization Scale (MPIS) was a first attempt to analyze the effects of post-traumatic cosmetic injuries on a Romanian population, being also a scale available to forensic doctors for an easy classification of scars or injuries even of those included in the category of cosmetic injury or slimming. MPIS takes into account not only morpho-functional and topographic aspects of the scars, but also several psychosocial factors involved in the individuals’ life [18,19]. It was conceived to measure the self-reported, subjectively assessed social support in patients with scars. Study participants responded to 15 statements listed, after self-evaluating on a Likert scale between 1, represents “I don’t agree”, and 5, “I totally agree”. The MPIS rating represents the summation, in a total score comprised between 15 and 75 points. The scale takes into account the self-consciousness of the presence of the scar, the gender of the victim, the morphological features of the scar, the negative influence of social interaction, and the impairment of the professional development of the patient [19]. The interpretation of MPIS was as follows: a score under 35 points—psychosocial internalization of the scar; between 35 and 54 points was considered aesthetic damage, and over 55 points—disfigurement.
- The Patient and Observer Scar Assessment Scale (POSAS) was proposed by Draaaijers et al. (2004) as a method for structured clinical evaluation of scar quality, reflecting the opinions of both the observer and the patient concerning the scars. POPSA is a consistent and reliable tool in the case of burn scars. Draaijers et al. showed that the instrument had alpha Cronbach coefficients between 0.76 and 0.69. POSAS points out scar characteristics such as vascularization, pigmentation, firmness, foldability, affected area, and scar height. POSAS is a standardized and validated tool in the evaluation of scars, also taking into account the patient’s symptoms related to scars, such as pain and itching, which were not taken into account in previous scales. In the study we used the PS subscale from POSAS where the recorded Cronbach alpha coefficient was 0.96 [18].
- Hopelessness Depression Symptom Questionnaire (HDSQ) is designed to measure the symptoms of hopelessness/depression. HDSQ contains 32 items that allows the examination of symptoms such as hopelessness and depression individually or in groups. In the HDSQ format each symptom is measured by a group of four items. Consequently, the instrument has eight subscales (Motivational Deficit—retarded initiation of voluntary responses, Interpersonal dependency, Psychomotor retardation, lack of energy, Apathy/anhedonia, Insomnia, Concentration difficulty and Suicidality), each of them including four items and measuring a different symptom of hopelessness/depression. Metalsky and Joiner (1997) reported Cronbach alpha coefficients between 0.70 and 0.93 for HDSQ. In our study the Cronbach alpha coefficient for HDSQ was 0.91 [19].
- Multidimensional Scale of Perceived Social Support (MSPSS) consists of 12 items that target three factors: family, friends, and significant people (in one’s life). Each item is structured according to these factors. The scales are anchored in such a way that the highest scores reflect the highest perceived social support. Zimet and Farley reported an SMSSP fidelity between 0.90 and 0.95 for the subscale and an alpha coefficient of 0.91 for the total SMSSP. In our study the alpha Cronbah coefficient was 0.98 [20].

### 2.3. Working Procedure

Study participants received information systematically and upon request, about the treatment that Could be given to reduce the inaesthetic appearance of scars, the benefits but also the complications that could occur from conservative or Surgical Therapies.

- Experimental design: (a) in this first part of the study, we captured the starting statistical indicators of the variables recorded for the experimental and control group with post-surgical scars. (b) In the second part of the study, we establish the fidelity coefficients for each scale used in the study as well as the correlations between the scales in the case of both the experimental and the control groups. (c) In the third stage of the research, the independent variable is represented by the classification of the participants in the two experimental lots: posttraumatic and postsurgical. Dependent variables are represented by, internalization of scars, hopelessness/depression, perception of social support, and evaluation of scars by the patient. The experimental design is unifactorial and the data were analyzed by the t-test for independent samples. (d) In the fourth stage of the research, we identify predictors which effectively support the estimation of the evolution of the dependent variable, the internalization of scars, in people with posttraumatic and surgical scarring. The predictive regression equation included the predictors of hopelessness/depression, scarring mode, PS Patient Scar Assessment) and the age at which scarring occurred.

### 2.4. Statistical Methods

For the variables included in the study, the K-S test calculated by the statistical package for the Social Sciences v.26 (SPSS, Chicago, IL, USA) to identify differences between the observed and theoretical distribution, showed that the participants’ results are not statistically significant (Z-Test) indicating a normal distribution (*p* > 0.05) that requires the use of parametric comparison techniques.

The quantitative variables presented above were statistically processed using the t test for independent samples taking into account the targeted unifactorial experimental designs. The fidelity of the research instruments was calculated with the Cronbach alpha coefficient. The correlation matrix (Pearson’s correlation coefficient) captures the association of relevant variables in the study. The predictive regression equation was implemented to identify the predictors that help to estimate the evolution of the dependent variable in the case of participants with scars in which these predictors were important both experimentally (statistically), and clinically. Preliminary analyses confirmed that the homogeneity assumption of linear regression was supported for all analyzes reported during the research. The diagnosis of multicollinearity showed that the chosen predictors did not correlate strongly with each other, the variance inflation factor (VIF) being under 10 and tolerance above 10. Multicollinearity was not identified in the case of the employed regression models, hence VIF and tolerance coefficients were not also presented.

## 3. Results

The 110 participants, 61 women (55.5%) and 49 men (44.5%), aged between 18 and 64 years (m = 40.33 ± 13) were distributed in two relatively homogeneous groups: experimental (*n* = 55) and controls (*n* = 55) as presented below.

In the experimental group, there were 30 women (54.5%) and 25 men (45.5%), aged between 18 and 64 years (mean = 39.98; SD = 13.53). Their declared marital state indicated 14 (25.5%) unmarried, 34 (61.8%) married, 4 (7.3%) divorced, and 3 (5.5%) widowed. From an educational point of view, 4 patients (7.3%) completed secondary education, 5 (9.1%) graduated vocational schools, 24 (43.6%) graduated high school, 15 (27.3%) were licensees of colleges/universities, and other 7 patients (12.7%) had master’s and doctoral degrees.

The control group included 55 participants, 31 women (56.4%) and 24 men (43.6%), aged between 19 and 64 years, (mean age = 40.69; SD = 14.68). Their declared marital state indicated 12 (21.8%) unmarried, 38 (69.1%) married, 1 (1.8%) divorced, and 4 (7,3%) widowed. From an educational point of view, 6 patients (10.9%) completed secondary education, 8 (14.5%) graduated vocational school, 18 (32.7%) graduated from high school, 13 patients (23.6%) were licensees of higher education institutions, and 10 patients (18.2%) has masters and doctoral degrees.

A first analysis of the recorded data of the participants included in the experimental group indicates a range of variability of ages at which the scars occurred between 6 and 61 years (mean = 30.32; SD = 14.05) and the time elapsed since the occurrence of scars was between 1 and 39 years (mean = 9.70; SD = 10.01). The scars occurred post-traumatically in the case of 51 (92.7%) participants, while only 4 (7.3%) reported surgically produced scars, but with a causal link with a previous traumatic event. Referring to the causative traumatic event, in 4 cases (7.3%) they were secondary to fall, 6 (10.9%) occurred due to aggression, 26 (47.3%) resulted after a road accident, 5 (9.1%) due to work accidents, in 9 cases (16.4%) there were household accidents, and in other 5 (9.1%) other causes.

Multiple scars were reported in 31 (56.4%) participants, and single scars in 24 (43.6%) while a preliminary analysis of their shape indicated 7 (12.7%) subjects with linear scars and 48 (87.3) with nonlinear scars. The size of the scars was between 1 and 60 cm (mean = 17.30; SD = 14.03) while in the case of the control group, the reported size was between 1 and 110 cm (mean = 13.30; SD = 17.49). Without significant values in their comparison (t = 1.323; *p* > 0.05), on the other hand, the scar area, measured in cm, indicates values between 1 and 70 cm (mean = 15.72; SD = 17.35) in the experimental group, while in the controls we recorded values between 1 and 55 cm (mean = 8.89; SD = 10.43) with a significant difference in the averages (t = 2.504; *p* < 0.01).

The adaptation time with the scars recorded between the experimental group and the controls indicated values between 0 and 10 years (mean = 2.75; SD = 2.52) in the experimental group and in the case of controls we recorded values between 0 and 7 (mean = 1.54; SD = 1.71), the *t*-test indicating a significant statistical value (t = 2.913; *p* < 0.004).

In Table 1 we present the frequency reported by the study participants assigned to the two groups, pertaining to how the mimicry as well as the symmetry of the body was affected. We noticed a more pronounced impairment in the case of the participants in the experimental group compared to the control group.

In Table 2 we present how patients consider that their social relations, their family relationships, as well as those with work colleagues, especially, were affected due to visible scars. In addition, we also included in the study a report on how the patients’ employment status has been affected.

Fidelity of the instruments used in study and inter-scale correlations.

The instruments used in the study (*n* = 110) have a high fidelity, which indicates stability, predictability, and accuracy. In Table 3 we present the averages, SD, and values of the alpha coefficients that are in the predictable area of the values recorded by the authors of the scales (see the tools section).

In the case of the experimental group (see Table 4), we observed an association between the psychosocial internalization of scars, measured with MPIS and the hopelessness (HDSQ) (r = 0.701; *p* < 0.001) that indicates a psychopathological orientation of patients. The obtained results highlight the relation with PS (r = 0.471; *p* < 0.001) where data were self-reported (painful scars, itching, color, stiffness, thickness, surface, etc.). In other words, a proximal and sufficient cause of depressive symptoms is the expectation that desirable results will not appear or highly aversive results will appear and no response in one’s own repertoire will change the probability of the occurrence of these results [21,22,23,24].

The analysis of the correlational parameters (see Table 5) presented in controls, MPIS, and the hopelessness depression (HDSQ) (r = 0.318; *p* < 0.05) indicates a psychopathological targeting of patients in the same line presented above. The internalization of scars measured with MPIS is associated with PS (r = 0.464; *p* < 0.001) that evaluates the results of the healing process of treated posttraumatic and surgical facial scars.

The results obtained in the investigation of the relationship between hopelessness depression were not done with PS (r = 0.043; *p* > 0.05). We note that the instruments that measure different aspects of scar integration have strong correlations between them, but not with the perception of social support (r = 0.054; *p* > 0.05) or with hopelessness depression (r = −0.441; *p* < 0.01) from which the patient dissociate themselves.

Starting from the fundamental assumptions, we compared a group of patients with traumatic (unexpected) scars with a group of patients with postsurgical scars (expected) depending, in relation, on how their lives were affected, respectively, and the probability of hopelessness.

The results presented in Table 6 support the model of the hopelessness depression subtype by the results recorded in patients with posttraumatic scars. Thus, motivational deficit (t = 2.521; *p* < 0.01), interpersonal dependence (t = 4.087; *p* < 0.001), psychomotor retardation (t = 4.287; *p* < 0.001), anergy (t = 4.692; *p* < 0.001), apathy/anhedonia (t = 5.765; *p* < 0.001), insomnia (t = 4.932; *p* < 0.001), difficulty in concentrating (t = 3.808; *p* < 0.001) and suicidal tendencies (t = 3.922; *p* < 0.001) indicate a greater vulnerability of patients with post-traumatic scars that appeared accidentally compared to patients of the control group who went through the stages of diagnosis and implicitly, scars appeared post surgically, which was expected. The recorded results indicate a greater vulnerability in the direction of learned helplessness and hopelessness of patients who have post-traumatically acquired scars even by the overall score at HDSQ (t = 4.927; *p* < 0.001).

Relationship between scar internalization and scar evaluation by the patient by the batch (experimental versus control).

We propose the term psychosocial internalization of scars, which we defined as the limit up to which a person can adapt with a scar in the social, family, and psychological environment. Internalization can also be defined as the habit with a scar that is dependent on the initial appearance of the person, age, and especially, the gender of the person. Starting from the presented assumption, we noted that patients with comparative post-traumatic scars versus controls, internalize scars (t = 4.991; *p* < 0.001) to a greater extent (see Table 7), but with psychopathological implications as we showed in Table 6.

Predictions regarding the prevalence, according to the internalization of the scars.

In the last stage of the study, we tried to identify the predictors that estimate the evolution, according to the internalization of the scars, and more precisely, which of the predictors are clinically effective. Preliminary analyses confirmed the compliance with the conditions of homogeneity and multicollinearity.

We postulate that hopelessness (HDSQ), patients’ appreciation of scarring (PO), age of scar production, and how scars are produced (surgically and post-traumatically) are important predictors in estimating scar internalization (MPIS).

Table 8 summarizes the statistical differences recorded following the dichotomization of the results according to the presence or absence of internalization (F(105) = 45.103; *p* < 0.001). Therefore, the coefficient of multiple determination, which is the percentage of the dispersion of scar internalization, explained by the joint action of the aforementioned predictors is R^2^ = 0.632 which indicates that the variables contribute in a proportion of 63.2% to the dispersion of scar internalization.

The t-significance test presented in Table 8 (excluding the interceptor/constant) states that predictors contribute significantly statistically to the estimation of scar internalization.

Predictors of hopelessness depression (β = 0.447; t = 6.592; *p* < 0.001) and the assessment of scars by the patients (β = 0.481; t = 7.083; *p* < 0.001) have the highest weight considering that they have the highest β value. In other words, predictors are relevant for the integration of scars, but not for further vulnerability from a psychopathological perspective. Predictors such as the age of scarring production (β = −0.152; t = −2.393; *p* < 0.01) and the way of scars production (β = −0.149; t = −2.428; *p* < 0.01) have a negative relationship with the internalization of scars, which orientates us toward a more effective integration of scars with the decrease of age (hypothetically they are easier to integrate in childhood).

The semi-partial correlation (rsp) of the internalization of the hopelessness depression scarring is rsp = 0.390 and the coefficient for determining the relationship between them is 15.21% which indicates a strong effect, the situation being similar in the case of the assessment of scars by patients (PO) where the dispersion of the internalization of scars is supported in a percentage of 17.55%. Predictors of scar production (2.07%) and scar production age (2.01%) have a cumulative effect.

## 4. Discussion

In the medical literature, aesthetic damage is a much-discussed issue because it results in a decrease in physical attraction or moral suffering, and having as consequence an aesthetic disfigurement associated with bodily harm. The evaluation of patients with scars represents a challenge for plastic surgeons, forensic specialists, and psychiatrists as well. Currently, in forensic practice, the aesthetic method of Greff and Hodin is still in use, a difficult procedure based only on morphological criteria [20,21,22,23]. The Patient and Observer Scar Assessment Scale (POSAS) is a questionnaire that assesses the quality of the scar from the perspective of both the observer and the patient [18].

The appearance of scars varies greatly depending on their location, individual and ethnic or racial characteristics of the patient, the nature of the trauma, and the conditions of wound healing causing itching, tension, and pain. Moreover, some patients experience psychological trauma, including anxiety, depression, post-traumatic stress disorder, loss of self-esteem, and stigmatization. All these problems may have important effects on the patient’s quality of life. Some authors consider that the aesthetic damage cannot be assessed because it also includes moral suffering [24], but other authors consider that the quality of life is affected because the success of post-traumatic social reintegration is conditioned [25].

In our study, we aimed to identify some of the factors that contribute to the psycho-social distress of the person with post-traumatic scarring. For this purpose, we employed a morphological scale (POSAS) in association with other scales such as MPIS, conceived to measure the self-reported, subjectively assessed social support in patients with scars [17], the Hopeless Depression Symptom Questionnaire (HDSQ), designed to measure hopelessness/depression [22], and the Multidimensional Scale of Perceived Social Support (MSPSS), that quantifies the self-reporting of subjectively assessed social support [23].

The research of Bianchi, Roccia, Fiorini, and Berrone supported our premises, evidencing that the highest score at PS indicates the most unfavorable scar imaginable [26]. Therefore, the patient’s opinion on the appearance of the scar (and not only of the doctor) is an indicator of how the patient will react in the future, on the one hand and on the other hand, to scar internalization (MPIS) and vulnerability to hopelessness. In our study, the t-test indicated a difference between the averages in favor of patients with post-traumatic scars, who consider scars much more disagreeable in comparison to subjects who acquired them post surgically.

Regarding the relationship between depression/hopelessness and traumatic versus postsurgical scars, Abramson et al. used the concept of “generalized hopelessness” when people exhibit negative results versus the expectation of hopelessness about several areas of life. According to Abramson et al. hopelessness depression occurs when the individual presents: (a) negative expectations regarding the occurrence of highly valued results and (b) expectations of inability to change the probability of the occurrence of these results (expectation of helplessness). The causes of generalized hopelessness are believed to produce severe symptoms of depression, while pessimism is associated with a limited number of symptoms with a low degree of severity [22]. We considered that the maladjusted attribution style in a specific field (such as in our study the production of scars in a traumatic or postsurgical way) also entails vulnerability to the symptoms of hopelessness/depression when an individual is faced with negative life events in the same instance (for example: social rejection following the appearance of scars).

Social support represents a person’s perception that he is cared for, valued, and that he or she is truly a part of a social network that supports him/her. The perception of social support has beneficial effects on mental and physical health, which is why we studied the relationship between it and the scars produced, post-traumatically (experimental group) and post surgically (controls). The analysis of our results regarding the perception of social support does not provide information to discern between the two groups in terms of the perception of family support (t = −0.640; *p* > 0.05), friends (t = −0.665; *p* > 0.05), significant persons (t = −0.791; *p* > 0.05), and the total score of MSPSS (t = −0.720; *p* > 0.05). We consider this important, given that social support often plays a significant buffer role [20,25]. Starting from this premises, we consider that social support is not perceived in a differentiated way by people with surgical or posttraumatic scars precisely because of the availability and psychological resources of the group members [27].

Between stress and the perception of social support there is a reverse relationship. Although, some well-known instruments employed for the evaluation of post-traumatic stress syndrome (PTSS) such as the Impact of Event Scale—Revised (IES-R) scale exist, they are mostly utilized for the evaluation of PTSS during the first month after the traumatic event. Due to the fact that scars need a longer time to finalize their appearance, our patients were evaluated after at least 6 months since the traumatic event, and without a baseline assessment we could not further use this scale for our study group.

Cutrona considered it important to take into account the availability and compatibility of social support as well as other characteristics of social support such as stability, proximity, and synchronization and between access to support and crisis situations [28]. Cohen considered that social support refers to “the provision of social support networks and psychological resources, deliberately, in order to benefit individuals to gain the skills necessary for adjustment under stressful conditions” [29].

The availability of social support depends on the characteristics of people with scars as well as on their communication skills. Therefore, personal and social characteristics that make communication impossible are likely to be associated with psychopathogenic effects such as ambivalence in emotional expression, repressive defensiveness, and fear of intimacy [25].

The investigation of the relationship between hopelessness depression and traumatic versus postsurgical scars in the context of the generalized hopelessness depression theory (Abramson, Metalsky, and Alloy) showed that the disadaptative attribution style in a specific field (for example, the production of scars in a traumatic or postsurgical way) also entails vulnerability to depression when a person perceives social rejection as a result of the occurrence of scars [22]. The results support the assumptions previously presented in patients with post-traumatic scars. Thus, motivational deficit, interpersonal dependence, psychomotor retardation, anergia, anhedonia, insomnia, difficulty concentrating, and suicidal tendencies indicate a sharp vulnerability in patients with post-traumatic scars that appeared contextually compared to patients who have scars after surgery.

We see an association between the psychosocial internalization of scars and that of hopelessness that can be an indicator of an orientation toward psychogenic depression. Feelings such as helplessness or hopelessness may increase the likelihood of the occurrence of these outcomes. In addition, the self-assessment of scars (painful scars, itching, color, stiffness, thickness, surface, etc.,) and association with hopelessness and depression indicate the expectation that the results of desirability, will not appear or will appear, in high aversive results and that no response in one’s own repertoire will change the probability of occurrence of these results [19,21].

The internalization of scars is dependent on the initial appearance of the person, the age, and, especially, the gender of the person, as well as, on their general state of health [30,31,32]. Our results show that people with post-traumatic scars are oriented toward the internalization of scars, but also according to their shape and size. In other words, the patient’s attitude to the appearance of the scar is an indicator of how the patient will react in the future and the vulnerability and hopelessness they may feel. The results obtained support previous claims that patients with post-traumatic scars consider their scars more disagreeable. The evaluation of aesthetic damage remains a component of forensic activity where the expert criterion is insufficiently outlined because of the weight of the subjective elements related to the traumatized victim. The difficulties stem from the fact that moral suffering cannot be objectively assessed, which entails impediments to the determination of the amount of compensation to be given by the competent authorities.

## 5. Conclusions

The investigation of the relationship between hopelessness/depression in traumatic versus postsurgical scars victims suggested that depressive symptoms indicate a sharp vulnerability of patients with post-traumatic scars in comparison to patients who have scars expected after surgery. MPIS indicated that the patient’s attitude to the appearance of the scar is an indicator for his future reaction and how vulnerable and/or hopeless he will feel.

We postulate that hopelessness, patients’ appreciation of scarring, the age of scar occurrence, and the modality of their production are important predictors for estimating scar internalization. In the prediction of the internalization degree of scars, the patient’s appreciation of size, shape, thickness, in association with hopelessness are relevant predictors and we noticed an effective internalization of scars in childhood and adolescence when, hypothetically, it is easier to integrate the scar with a patient’s favorable body image.

## Figures and Tables

**Table 1:** Reported frequency of scars in the case of the two groups, depending on the affected area.

Group		Mimic Damage		Impaired Body Symmetry	
Experimental	Of	31	56.4%	30	54.5%
	Right away	24	43.6%	25	45.5%
Controls	Of	13	23.6%	12	21.8%
	Right away	42	76.4%	43	78.2%

**Table 2:** Reported frequency of perceived relationship impairments due to scars.

Group		Social Withdrawal		Family Relationships		Colleagues Work		Health Insurance		Job	
experimental	Of	27	49.1%	21	38.2%	26	47.3%	36	65.5%	19	34.5%
	Right away	28	50.9%	34	61.8%	29	52.7%	19	34.5%	36	65.5%
controls	Of	5	0.9%	4	7.3%	6	10.9%	34	61.8%	4	70.3%
	Right away	50	90.1%	51	927%	49	89.1%	21	38.2%	51	92.7%

**Table 3:** Mean, standard deviation, ranks, and alpha coefficients for the scales included in the study.

Scale	Media	SD	Minimum Value	Maximum Value	Alpha Coefficients
1. MPIS	41.77	13.90	19	65	0.89
2. MSPSS	5.16	1.96	0	7	0.98
3. HDSQ	20.51	24.45	0	142	0.91
4. PS-POSAS	30.90	17.95	7	70	0.96

Legend: MPIS-Mekeres Psychosocial Internalization Scale; MSPSS: Multidimensional Scale of Perceived Social Support; HDSQ: Hopelessness Depression Symptom Questionnaire; PS-POSAS: Patient Scale-Patient and Observer Scar Assessment Scale; SD: standard deviation.

**Table 4:** Correlations between the scales in the case of the experimental group.

Scales	1	2	3	4
1. MPIS	1			
2. MSPSS	−0.091	1		
3. HDSQ	0.701	−0.137	1	
4. PS-POSAS	0.677	−0.104	0.471	1

Legend: MPIS: Mekeres Psychosocial Internalization Scale; MSPSS: Multidimensional Scale of Perceived Social Support; HDSQ: Hopelessness Depression Symptom Questionnaire; PS-POSAS: Patient Scale-Patient and Observer Scar Assessment Scale.

**Table 5:** Correlations between the scales in the case of the control group.

Scales	1	2	3	4
1. MPIS	1			
2. MSPSS	0.054	1		
3. HDSQ	0.318	−0.441	1	
4. PS-POSAS	0.464	−0.094	0.043	1

Legend: MPIS: Mekeres Psychosocial Internalization Scale; MSPSS: Multidimensional Scale of Perceived Social Support; HDSQ: Hopelessness Depression Symptom Questionnaire; PS-POSAS: Patient scale-Patient and Observer Scar Assessment Scale.

**Table 6:** Batch comparisons (experimental versus control) to HDSQ.

Hopelessness Depression Subtypes	Lot	N	M	SD	t	*p*
Motivational deficit	experimental	55	5.40	10.48	2.521	0.01
	Controls	55	1.72	2.61		
Interpersonal addiction	experimental	55	3.60	3.38	4.087	0.001
	controls	55	1.34	2.29		
Psychomotor retardation	experimental	55	3.01	3.25	4.827	0.001
	controls	55	0.67	1.54		
Anergia	experimental	55	3.18	3.21	4.692	0.001
	controls	55	0.90	1.60		
Apathy/anhedonia	experimental	55	4.50	3.60	5.765	0.001
	controls	55	1.20	2.26		
Insomia	experimental	55	4.74	3.61	4.932	0.001
	controls	55	1.80	2.55		
Difficulty concentrating	experimental	55	4.61	4.21	3.808	0.001
	controls	55	2.05	2.66		
Suicide/Trends	experimental	55	1.87	2.55	3.922	0.001
	controls	55	0.38	1.19		
Depression of total hopelessness	experimental	55	30.94	27.79	4.927	0.001
	controls	55	10.09	14.59		

**Table 7:** Batch comparisons (experimental versus control) to MPIS and PS.

Scales	Group	N	M	SD	t	*p*
MPIS	experimental	55	47.76	13.65	40.991	0.001
	controls	55	35.78	11.42		
PS-POSAS	experimental	55	36.90	18.70	30.705	0.001
	controls	55	24.90	15.07		

Note: MPIS: Mekeres Psychosocial Internalization Scale; PS-POSAS: Patient Scale-Patient and Observer Scar Assessment Scale.

**Table 8:** Predictive multilinear regression equation in patients with scarring according to psychosocial internalization (MPIS).

Model	R	R^2^		F	f 1	*p*
1	0.795	0.632		45.103	4	0.001
Model	Non-standardized coefficients		Standardized coefficients	t	*p*	CS r_sp_
	B	IT	Beta (b)	10.672	0.001	
(Constant)	34.643	3.246				
Hoplessness depression	0.254	0.039	0.447	6.592	0.001	0.390
How scars were produced	−40.244	10.748	−0.149	−2.428	0.01	−0.144
PS	0.373	0.053	0.481	7.083	0.001	0.419
Age of scars apparition	−0.137	0.057	−0.152	−2.393	0.01	−0.142

Legend 1: Predictors: (Constant), hopelessness depression, scarring mode, PS: Patient scale-Patient and Observer Scar Assessment Scale, age of the scar production; Legend 2; CS/rsp—semi-part correlation.

## Data Availability

Our data are available on https://data.mendeley.com/datasets/tj4t84rpvg/1, accessed on 6 March 2022.
